# Assessing genome conservation on pangenome graphs with PanSel

**DOI:** 10.1093/bioadv/vbaf018

**Published:** 2025-03-05

**Authors:** Matthias Zytnicki

**Affiliations:** Unité de Mathématiques et Informatique Appliquées, INRAE, 31 326 Castanet-Tolosan, France

## Abstract

**Motivation:**

With more and more telomere-to-telomere genomes assembled, pangenomes make it possible to capture the genomic diversity of a species. Because they introduce less biases, pangenomes, represented as graphs, tend to supplant the usual linear representation of a reference genome, augmented with variations. However, this major change requires new tools adapted to this data structure. Among the numerous questions that can be addressed to a pangenome graph is the search for conserved or divergent genes.

**Results:**

In this article, we present a new tool, named PanSel, which computes a conservation score for each segment of the genome, and finds genomic regions that are significantly conserved, or divergent. PanSel can be used on prokaryotes and eukaryotes, with a sequence identity not less than 98%.

**Availability and implementation:**

PanSel, written in C++11 with no dependency, is available at https://github.com/mzytnicki/pansel.

## 1 Introduction

Genome diversity has long been studied for genetic diseases, genome improvement of species of agricultural interest, among others. With the advent of new, long, almost errorless, reads, it is now possible to produce several telomere-to-telomere haplotypes for complex eukaryotes. These haplotypes, if they include the diversity of the population of interest (usually, a species), may be compared, leading to pangenomic studies (The Computational Pan-Genomics Consortium 2018). A pangenome can be efficiently stored into a dedicated data structure, which is usually a variation graph (Garrison *et al.* 2018). This graph contains so-called *segments*, which are sequence chunks found in at least one haplotype, and “paths,” which are lists of segments (possibly in reverse-complement) representing haplotypes. A given segment, if it belongs to several paths, represents a homologous sequence shared by the haplotypes.

Although pangenomic studies have been published for almost two decades in prokaryotes, they are relatively new for multicellular eukaryotes, and changing the framework from a genome supplemented with variations to a variation graph is no simple task. Notably, it requires adapting all the tools, which were developed for the previous framework, and there are numerous. It is, for instance, crucial to be able to assess the conservation, or the divergence, of genome “loci,” i.e. sequences that evolve slower (or faster) than the expected natural drift. Several methods have been developed for inter-species analyses with a reference genome for each organism, and they include phastCons (Siepel *et al.* 2005), GERP and GERP++ (Davydov *et al.* 2010), and phyloP (Pollard *et al.* 2010). They use a multiple sequence alignment, and a phylogeny, in order to estimate the length of the branches of the tree at each position of the genome alignment. These metrics are then widely used, available in Genome Browsers (Raney *et al.* 2024), and can be used to check whether a gene of interest is conserved through evolution.

To date, there is no such method for intra-species conservation studies, and inter-species tools cannot be used as is. For instance, the phylogenetic tree is usually not known, or even not defined, between individuals of the same species, because inbreeding may be frequent. Some methods do find conserved regions, usually using k-mers, such as Corer (Schulz *et al.* 2022) or (Lee *et al.* 2023), and cluster them into “cores” or “pan-conserved segment tags.” Yet, finding divergent regions, or assessing the conservation of a gene of interest, is not possible.

Our aim is to provide a simple tool that could be applied by consortia that produce variation graphs, in order to assess the conservation of each point of the genome, so that users could check whether their genes of interest, or their genomic regions of interest in general, are under selection pressure, or accumulating variations.

We wanted to leverage the variation graph because it is now the standard model for representing diversity. Yet, the base-level multiple alignment—which tentatively puts in the same column the nucleotides that evolved from the common, ancestor nucleotide—is not available in the variation graph. Finding a conservation value for each nucleotide is thus not directly applicable. So, we depart from the base-level resolution, which is not easily accessible using a pangenome graph. Instead, we use a sliding windows of fixed size (e.g. of size 1 kb), which is enough to detect conserved and divergent regions. Second, we estimate the diversity by computing the Jaccard index (as defined by ODGI (Guarracino *et al.* 2022)) between each pair of paths in each window. Last, significantly under- and over-conserved windows are detected by fitting a mixture model.

## 2 Implementation

PanSel is a C++ tool that reads a GFA file representing one chromosome (it thus cannot, in its current version, deal with chromosomal translocations). A detailed description of the implementation is given in Supplementary Section 1. Briefly, the graph is divided into bins of size s, a parameter provided by the user, and a conservation score is computed for each bin. To do so, PanSel tries to detect conserved segments, shared by each path (named *boundary segments* hereafter) distant by s nucleotides on the reference path. The sub-path for each path is then extracted between each pair of consecutive boundary segments. The average (weighted) Jaccard index (Guarracino *et al.* 2022) is then computed between each pair of sub-paths in the same bin.

**Figure 1.**
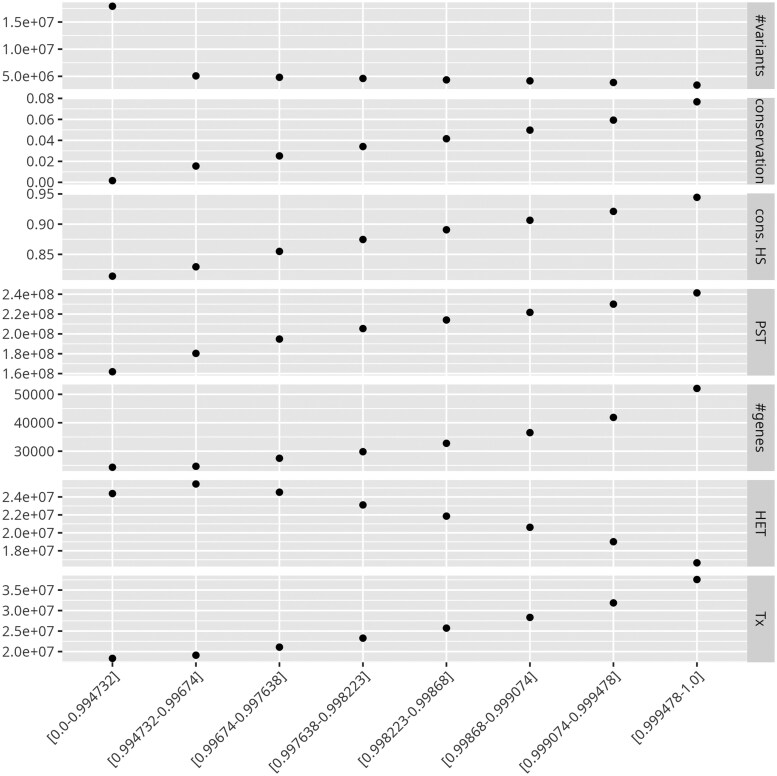
Correlation between conservation and several annotations. Each column represents a bin. The bins on the left include the most divergent regions, whereas the ones on the right include the most conserved regions. The Jaccard index intervals are provided in the *x* labels. For each bin, we computed the number of overlapping structural variants (first row), the average vertebrate conservation, the average human conservation, the number of pan-conserved segment tags, the exonic coverage, the predicted heterochromatin coverage, and the predicted actively transcribed coverage (last row).

Since the Jaccard index belongs to the [0,1] interval, we map it $R_{+}$ using the −log  function. The distribution is then fitted with a simple mixture model (see Figs 1–3 in Supplementary Data). The most conserved part follows a Gaussian distribution. However, we observe a heavy tail on the right-hand side of the distribution, which represents divergent sequences, and is not compatible with a Gaussian distribution. We model it using a log-normal distribution, and finally fitted the whole distribution with the average of the two previously found distributions.

**Figure 2.**
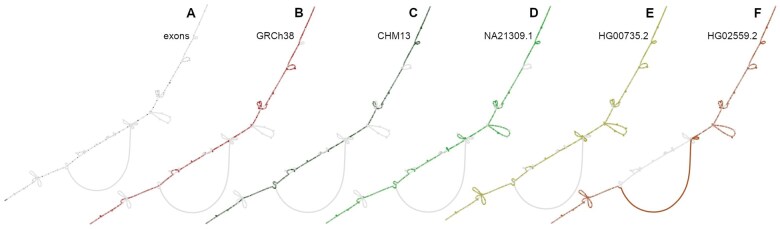
Bandage (Wick *et al.* 2015) captures of different paths of *NBPF20*. A: Grey lines show all the paths, and black dots show exons of *NBPF20*, as annotated in GRCh38. B: The highlighted path represents the reference assembly GRCh38. C: The highlighted path represents the telomere-to-telomere assembly CHM13. D–F: The highlighted paths represent haplotypes of NA21309.1 (Maasai), HG00735.2 (Puerto Rico), and HG02559.2 (Barbados), respectively.

## 3 Results

We applied PanSel with three sliding window sizes (1000, 10 000, and 100 000) on the Draft Human Pangenome (Liao *et al.* 2023). We took the 11 March 2022 release, computed by MiniGraph-Cactus (Hickey *et al.* 2024).

In our computer (Intel^®^ Xeon^®^ CPU E5-2687W v4 @ 3.00 GHz running Debian x86_64 6.1.76-1), PanSel took <22 min, and not more than 20GB RAM, for each chromosome (run on a single thread per chromosome).

### 3.1 Comparison with other annotations

First, we discarded the regions that overlapped with known gaps (see Supplementary Section 1.6). We then divided the regions into eight categories of equal sizes, with the most divergent regions being in the first bin, and the most conserved regions being in the last bin. For the sake of space, we will only present results for a window size of 1 kb, but results for other windows sizes follow the same trend and are presented in Supplementary Section 3.1.

We compared each bin with several available annotations. First, we counted the number of structural variants that overlapped with each bin. As seen in Fig. 1 (first row), the number of variants decreases with the bin number, and the most divergent bin overlaps with significantly more known variants.

We computed the average conservation score, provided by PhyloP (Pollard *et al.* 2010) using 100 vertebrates, on each bin. The second row of Fig. 1 shows that the conservation increases with the bin number. This confirms that genomic regions that are more conserved in human are also more conserved in vertebrates.

We also computed the PhastCons (Siepel *et al.* 2005) score (which took several days to complete), based on a human pangenome multiple alignment, and provided the average score in the third row. The number of pan-conserved segment tags (Lee *et al.* 2023) is given in the fourth row. The different methods provide concordant results. We tried to use Corer (Schulz *et al.* 2022), but it did not complete in 4 days with 80 CPUs.

We compared the bins with the location of known protein-coding exons, provided by GenCode (Frankish *et al.*, 2021), and computed the number of exonic nucleotides found in each bin. As seen in the fifth row of Fig. 1, conserved regions contain more coding exons, as expected.

Last, we extracted the annotation computed by ChromHMM (Ernst and Kellis 2012). Briefly, ChromHMM splits the genome into 100 categories (Vu and Ernst 2022), based on ChIP-Seq data. These categories are associated with different states of the chromatin (e.g. “actively transcribed,” or “Tx”). Each state may have different levels (e.g. there are eight “Tx” levels) and we merged these levels in this study. Here, we focused on the “Tx” and the “HET” (for heterochromatin) states, but all the results can be found in Supplementary Section 3.2. As seen in the sixth row of Fig. 1, heterochromatin-rich regions are more divergent. The last row shows that actively transcribed regions are more conserved, as expected.

### 3.2 Study of a particular gene

We wanted to showcase the use of PanSel on human genes. We extracted the coding genes located inside the most divergent regions. Among them, we found *ANKRD30A*, *BRF1*, and *NBPF20*. The example of the latter gene is presented in Fig. 2, while the others are presented in Supplementary Data, Section 3.3.

*NBPF20* belongs to the neuroblastoma breakpoint family (Heft *et al.* 2020, Glunčić *et al.*, 2023), which recently expanded, especially in humans. Some genes may thus undergo neo- or sub-functionalization and accumulate variations. Figure 2 shows the human pangenome graph, restricted to this gene. In Fig. 2A, we highlighted in black the exons of the gene (as annotated in GRCh38). In Fig. 2B and C, we highlighted the GRCh38 and CHM13 paths, respectively. The last three subfigures show haplotypes of different people. These figures pinpoint the wealth of SVs in the diversity. Some of them may alter the transcript: some exons are skipped and are thus missing in the final transcripts; some exons may also be added, since new genetic material is present. SVs in this gene family, which include higher order repeats, are well documented and may be linked with cognitive disabilities, among others. Proving that this gene is evolving rapidly in humans, taking into account both SNPs and SVs, has not been done before and highlights the usefulness of PanSel.

### 3.3 Conserved and divergent regions

Another use of PanSel is the study of regions that are significantly conserved and divergent. We used a *P*-value of 5% for a window size of 10 000 (a window size of 1000 is usually too small to capture the full extent of a gene). We found ∼79 and 162 Mbp of conserved and divergent regions, respectively. Figs 13–15 in Supplementary Data show the distribution of conserved and divergent regions on the autosomes. As expected, divergent regions accumulate near the telomeres, while convergent regions are located inside chromosomes.

We then extracted the overlapping genes (4861 and 12 286 genes in conserved and divergent regions, respectively) and looked for enriched gene ontologies using gProfiler (Kolberg *et al.* 2023).

Full results are provided in Supplementary Section 3.4. Briefly, conserved regions are related to very general molecular functions (e.g. protein binding), biological processes (e.g. multicellular organism development), or cellular compartment (e.g. nucleoplasm).

Divergent regions are related to more specific molecular functions (e.g. antigen binding), biological processes (e.g. adaptive immune response), or cellular compartment (e.g. immunoglobulin complex), which are related to immunity.

This would suggest that basic molecular functions are located in conserved regions, whereas immunity-related genes are located in divergent regions, as expected.

### 3.4 Other uses

We run PanSel on graphs generated with another tool, PGGB (Eizenga *et al.*, 2020). Results, provided in Fig. 10 in the Supplementary Data, show moderate correlation between both tools (between 0.42 and 0.84 Pearson correlation). Graphs produced by PGGB yield less conserved scores, possibly because more divergent scaffolds are included in the graphs.

We also tested PanSel on a bacteria, *Myxococcus xanthus*. First results, provided on Fig. 18 of Supplementary Results, show a good correlation between conservation and gene coverage.

We showed, in Fig. 4 of Supplementary Data, that PanSel provide reliable results, up to an estimated sequence similarity of 98%.

## Supplementary Material

vbaf018_Supplementary_Data

## Data Availability

The pipe-line used to produce the results can be retrieved from https://github.com/mzytnicki/pansel_paper.
