# Supplementary material for: Assessing genome conservation on pangenome graphs with PanSel
Source: Bioinform Adv. 2025 Mar 5;5(1):vbaf018. doi: 10.1093/bioadv/vbaf018 (PMC11908644; doi:10.1093/bioadv/vbaf018)
Supplement: vbaf018_Supplementary_Data [file vbaf018_supplementary_data.pdf]

PanSel – Supplementary Data

1 Description of the method

1.1 GFA parsing

PanSel reads the `S`, `P`, and `W` lines of the GFA file. All the other lines are skipped. It stores the lengths of the segments (but not the nucleotidic sequences), and the paths (as lists of segments). In some GFA files, variations graphs have been enriched with additional variations, some of them being named `_MINIGRAPH_.sXXXX` (where `X` is a digit) by the MiniGraph-Cactus pipe-line. These lines are skipped.

1.2 Boundary segments

In this step, PanSel finds all the segments that are included in all paths. These segments represent conserved sequences. The number of paths can be provided by the user. Alternatively, PanSel can detect it. The *boundary* segments that are included in all the paths are then stored.

1.3 Sliding windows

PanSel then follows the nodes of the reference path (provided by the user). It scans each segment, which can be represented as a genomic interval $[a, b]$ on the reference path. It stops at the first conserved segment, A representing interval $[a, b]$. This is the first boundary segment. From there, it tries to find the next boundary segment, B, such that the distance between A and B is approximately s, the size of the user-defined sliding window. More formally, we are looking for, B, representing interval $[c, d]$, such that $c - b \leq s \leq d - a$.

There may be no such boundary segment: the position of the ending sliding window may contain variations. In this case, we look for the next boundary segment, and record that the size of the sliding window will be larger than expected.

We then proceed to the next interval.

1.4 Computing the weighted Jaccard index

We extract the sub-paths of each path between each pair of selected, consecutive boundary segment. Given two sub-paths, stored as two sets of segments A and

B, the Jaccard index is the ratio between the size of the intersection of the segments, divided by the size of the union:

$$\frac{|A \cap B|}{|A \cup B|}$$

However, some segments are longer than others, and a SNP should "weight" less than a structural variation of, say, 100 base pairs. Following the method defined in ODGI, we use the following formula as the weighted Jaccard index (where n is a segment, and $|n|$ is its length):

$$\frac{\sum_{n \in A \cap B} |n|}{\sum_{n \in A \cup B} |n|}$$

For each sliding window, we compute the average weighted Jaccard index between all pairs of sub-paths.

1.5 Implementation analysis

PanSel stores all the nodes for every path. It then iterates over the windows, and extracts the nodes of every path. For every window, and every pair of paths, it then compares the nodes of the sub-paths. The method is thus linear with respect to the sizes of the paths (computed as the number of nodes in each path), and quadratic with respect to the number of paths. In principle, adding more paths (and individuals) would then render the method impractical. To speed up the comparison step, we order the nodes, and perform a simple sweep on these nodes. This makes the algorithm very fast in practice.

The method takes less than 22 minutes for the longest chromosome of the draft human pangenome, which includes 94 haplotypes, plus 2 other paths (GRCh38 and CHM13) for a window size of 1k. The time decreases to less than 10 minutes for a 100k window size. The graph includes 1,664,534 nodes for the longest chromosome, and 91,151,644 nodes for all the autosomes, which is probably the largest eukaryotic pangenome graph, considering the number of paths. The method is thus expected to scale well in practice.

1.6 Fitting the average weighted Jaccard index

We first compute the distribution of the average weighted Jaccard index per region. Since the index is in the $[0, 1]$ interval, we translate it to the $\mathbb{R}_+$ using the $-\log$ transformation. We compute the mode m, and extract the region corresponding to $x \in [0, m]$. We add the symmetric of this distribution, and fit a normal distribution. We also fit the whole distribution with a log-normal, and plot the average of the two distributions.

The p-value can then be computed directly from the mixture model. In order to find the significantly conserved (resp. divergent) regions, we take the extreme 5% of the normal (resp. log-normal) distribution. The threshold can be modified by the user.

The fits are plotted in Figures 1, 2, and 3.

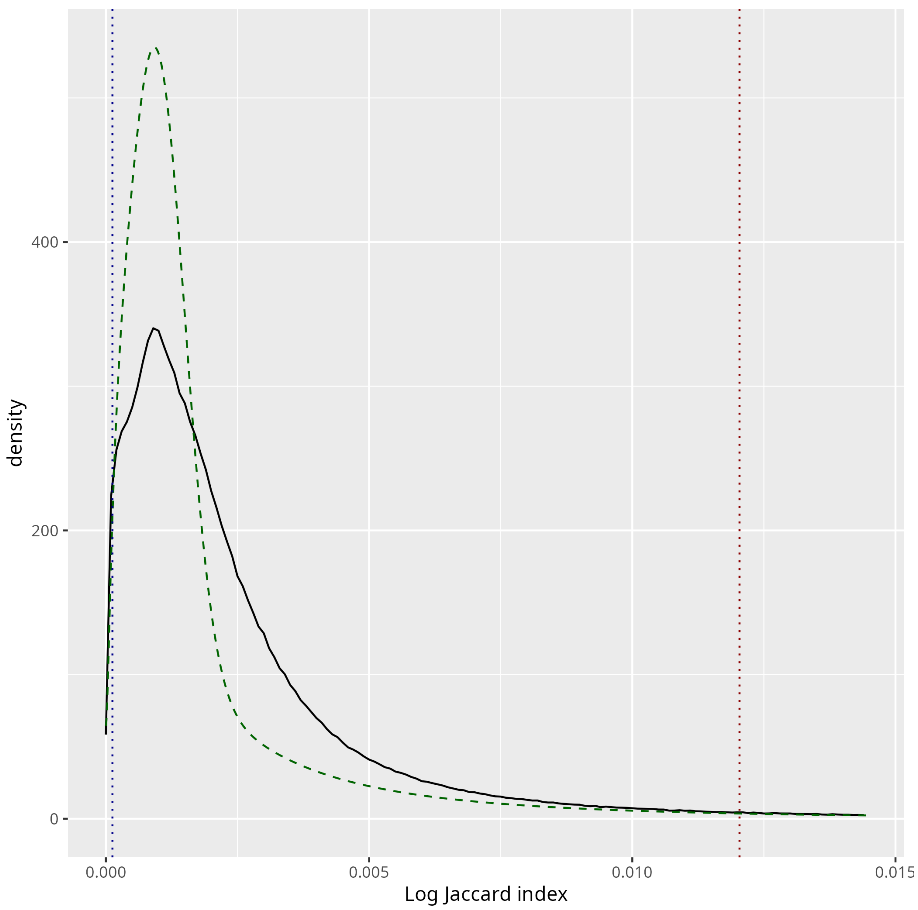

Figure 1: Distribution of the average weighted Jaccard index, in solid black line, for a window size of 1000. The fit is in dashed green, the threshold for significantly conserved (resp. divergent) regions is in dotted blue (resp. red).

1.7 Splitting data to different conservation strata

The script `divideRegions.py` (see the corresponding Github page) reads the output of Pansel. It first removes regions that are located in known gaps of the GRCh38.

In some cases, PanSel cannot find boundary segments distant by s nucleotides (s being given by the user). This is usually due to large insertions, deletions, or variations. In this case, PanSel extends the size of the window, in order to find the next boundary segment. If the window is too large (1.5 times the size of the window size given to PanSel), the script removes these regions. In the 1kb data daset, 438,939,840bp are located in these regions, and 49,685bp are in gaps, leaving 2,574,066,607bp for the analysis. Then, it ranks

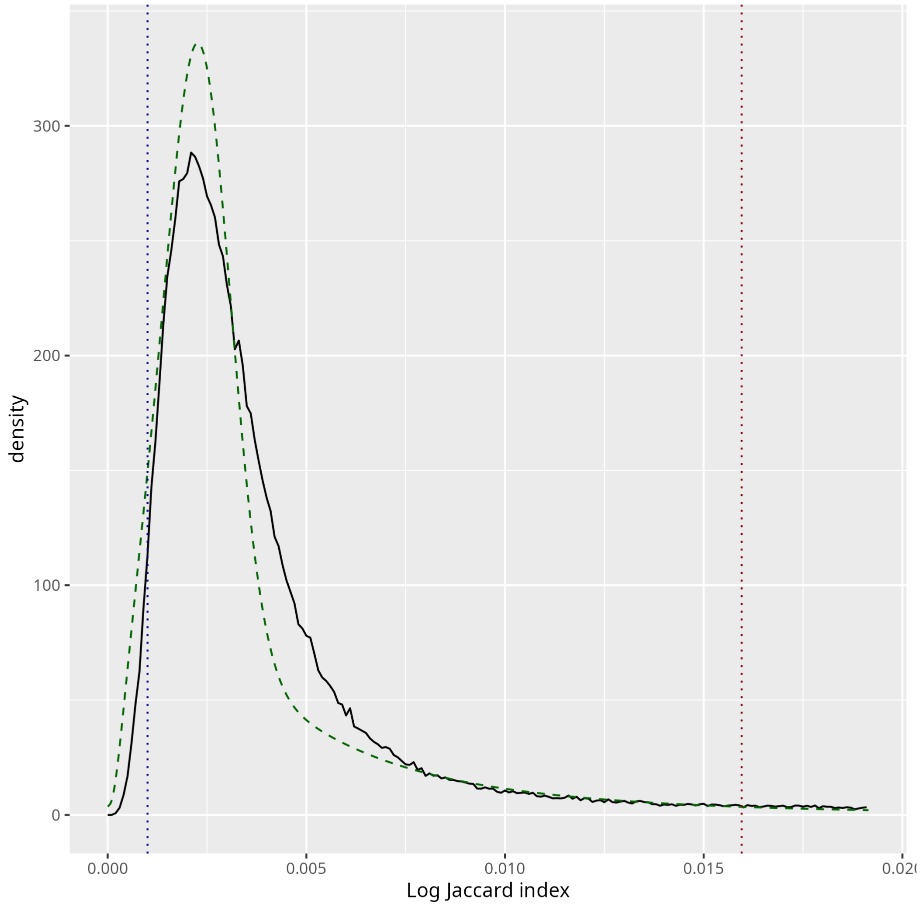

Figure 2: Distribution of the average weighted Jaccard index, in solid black line, for a window size of 10,000. The fit is in dashed green, the threshold for significantly conserved (resp. divergent) regions is in dotted blue (resp. red).

the remaining regions based on the Jaccard index, and splits them into strata of (roughly) identical sizes.

1.8 Estimating the conservation range where PanSel works best

As explained in Section 1.8, PanSel cannot always find boundary segments distant by s nucleotides. The conservation of these regions are thus less accurately assessed. Larger regions may be considered as an evidence of sub-optimal results.

Jaccard index do not model structural variations such as chromosomal re-

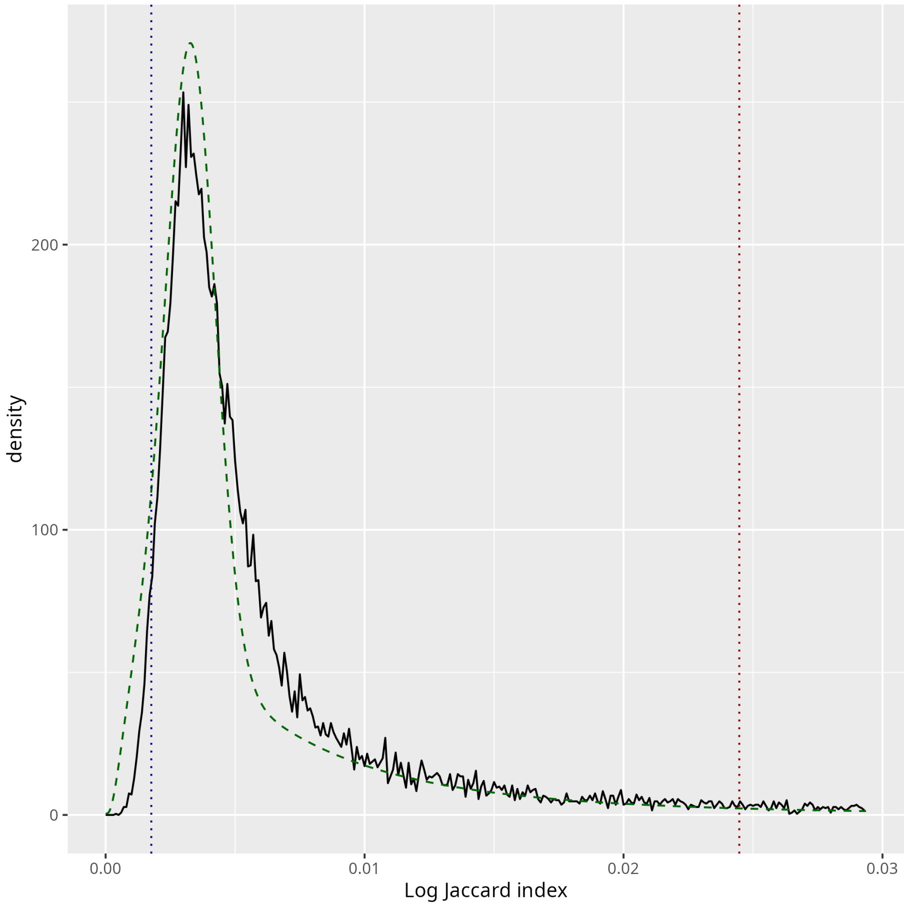

Figure 3: Distribution of the average weighted Jaccard index, in solid black line, for a window size of 100,000. The fit is in dashed green, the threshold for significantly conserved (resp. divergent) regions is in dotted blue (resp. red).

location or repetition. However, it is a good proxy for small variations. We plotted, in Figure 4, the Jaccard indices computed by PanSel with a window size of 10kb. On the left, we provided the scores for the windows with roughly the expected size ($\leq$ 15kb), and the larger windows on the right ($>$ 15kb). The median Jaccard index (in the violin plot) of the windows with larger sizes is at 0.975. We can thus estimate that regions with 98% identity can be correctly analyzed.

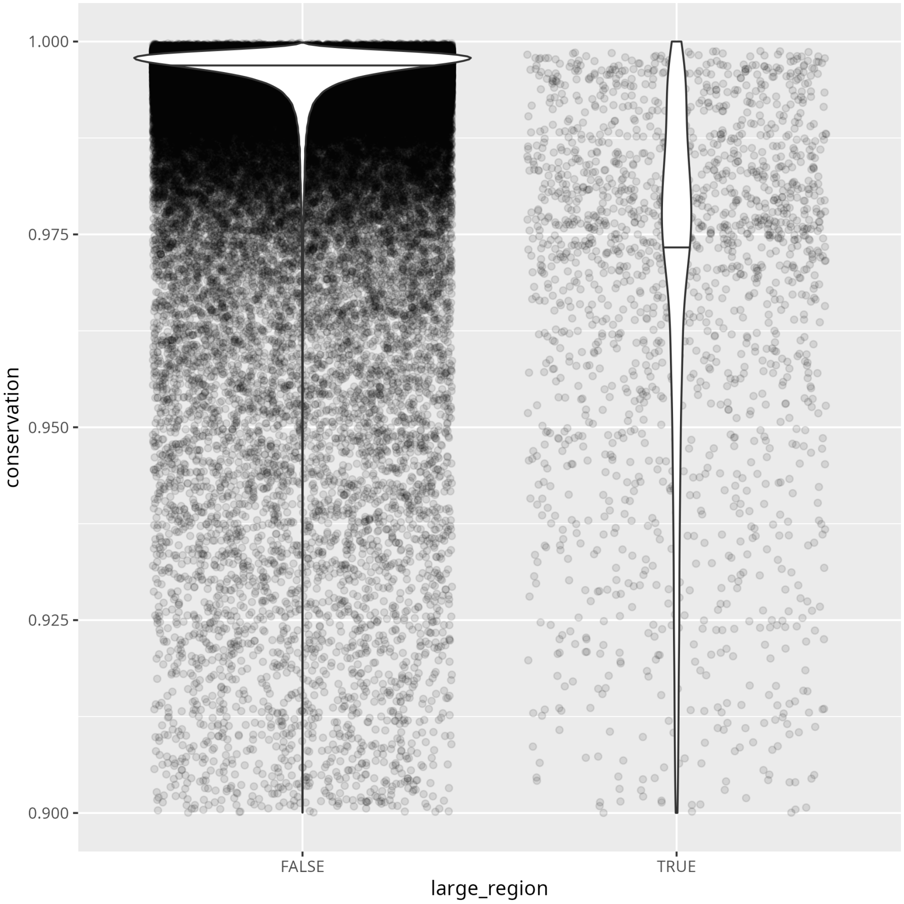

Figure 4: Distribution of the average weighted Jaccard index for windows with the expected sizes (on the left) and windows with larger sizes (on the right). For readability, only scores not less than 0.9 are plotted.

2 Data used

2.1 Human data

2.1.1 Pangenome graphs

The pangenome graphs were downloaded from the Human Pangenome initiative:
`https://s3-us-west-2.amazonaws.com/human-pangenomics/index.html?prefix=pangenomes/scratch/`

For MiniGraph-Cactus, we chose the `2022_03_11_minigraph_cactus` release, and downloaded one `vg` file per chromosome. Each file was then converted to GFA format using `vg view`.

For PGGB, we chose the `2021_07_30_pggb` release.

We chose GRCh38 as a reference, since many annotation are available.

2.1.2 Genome annotations

- Assembly gaps:
  `https://hgdownload.soe.ucsc.edu/goldenPath/hg38/bigZips/latest/hg38.agp.gz`

- List of structural variants from dbVar:
  `https://ftp.ncbi.nlm.nih.gov/pub/dbVar/data/Homo_sapiens/by_assembly/GRCh38/vcf/GRCh38.variant_call.all.vcf.gz`

- 100 vertebrate conservation using PhyloP:
  `https://hgdownload.soe.ucsc.edu/goldenPath/hg38/phyloP100way/hg38.phyloP100way.bw`

- Genome annotation:
  `https://ftp.ebi.ac.uk/pub/databases/gencode/Gencode_human/release_44/gencode.v44.annotation.gtf.gz`

- ChromHMM annotation:
  `https://public.hoffman2.idre.ucla.edu/ernst/2K9RS/full_stack/full_stack_annotation_public_release/hg38/hg38_genome_100_segments.bed.gz`

- Multiple alignent of human genome in HAL format (used by PhastCons):
  `https://s3-us-west-2.amazonaws.com/human-pangenomics/pangenomes/scratch/2022_03_11_minigraph_cactus/hprc-v1.1-mc-grch38-full.hal`

- Pan-conserved segment tags:
  `https://dna-discovery.stanford.edu/publicmaterial/datasets/pangenome/pst-31mer.grch38.bed.gz`

2.2 *Myxococcus xanthus* data

We used the data set provided as Supplementary Data of BubbleGun [1] for the genomes. The annotation was retrieved from `https://ftp.ncbi.nlm.nih.gov/genomes/all/GCA/000/012/685/GCA_000012685.1_ASM1268v1/GCA_000012685.1_ASM1268v1_genomic.gtf.gz`

3 Results

3.1 Scores for the other window sizes

This part gives the same results as Figure 1 in the main document, with different window sizes.

3.1.1 Window size of 10k

For 10k window size, results are very similar to 1k size.

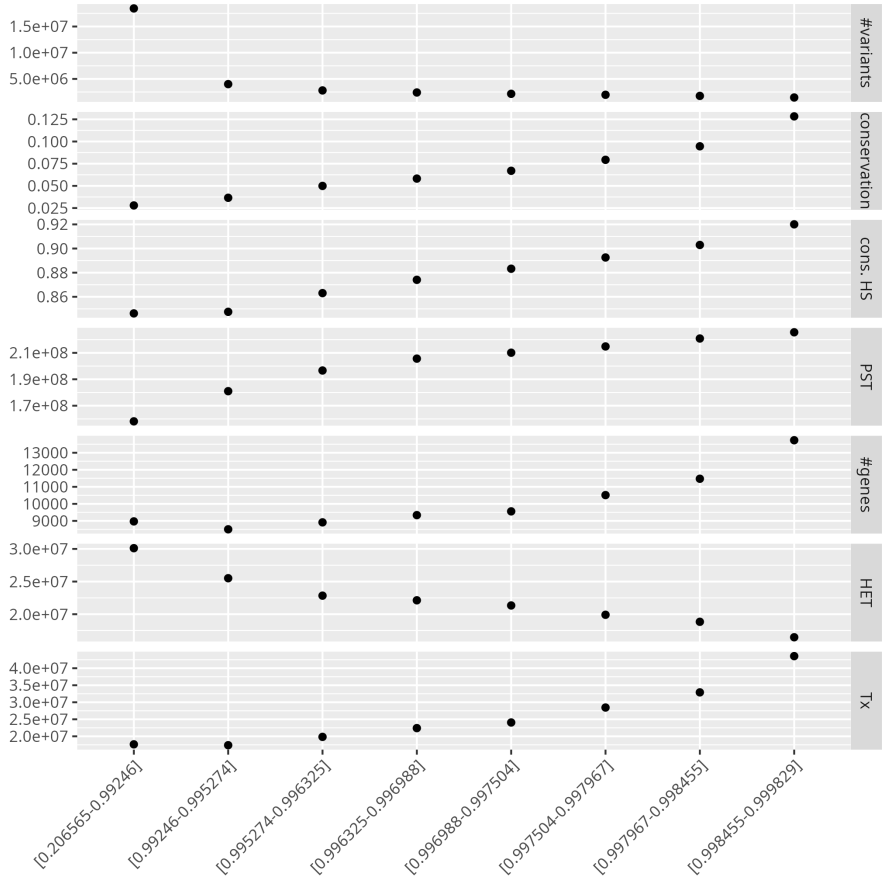

Figure 5: Results for window size of 10k. See Figure 1 in the main document for a detailed legend.

3.1.2 Window size of 100k

At 100k, the signal is less clear, probably because windows are too large, and include very diverse types of regions.

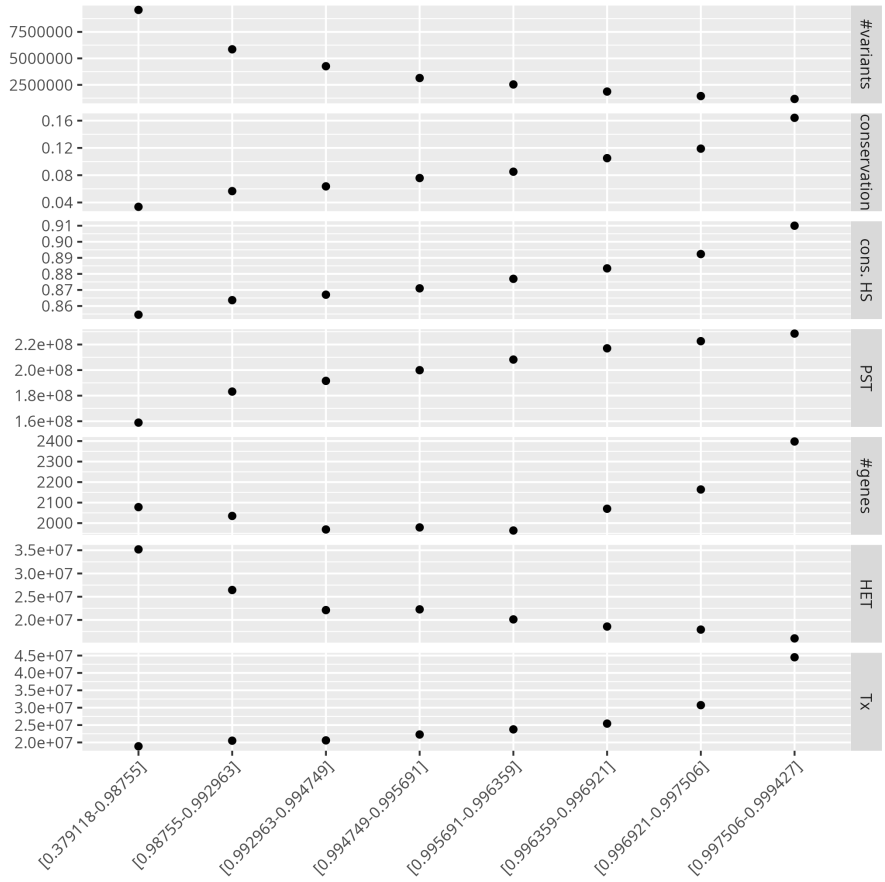

Figure 6: Results for window size of 100k. See Figure 1 in the main document for a detailed legend.

3.2 ChromHMM full results

This part gives the coverage for all ChromHMM categories. The categories are:

- Acet: acetylations
- BivProm: bivalent promoters
- DNAse: DNase I hypersensitivity
- EnhA: active enhancers
- EnhWk: weak enhancers
- GapArt: assembly gaps and alignment artifacts
- HET: heterochromatin
- PromF: Flanking promoters
- Quies: repressive or inactive
- ReprPC: polycomb repressed
- TSS: transcription start site
- Tx: strong transcription
- TxEnh: transcribed enhancers
- TxEx: transcript exons
- TxWk: weak transcription
- ZNF: zinc finger

3.2.1 Window size of 1k

Figure 7 clearly shows that repressed states, including HET, Quies, and ReprPC, tend to co-localize with divergent regions. It is also the case for Acet states, which is a weak mark of promoter or enhancers.

On the other hand, active states, including BivProm, EnhWk, PromF, TSS, TxEnh, TxEx, TxWe, and ZNF, tend to co-localize with conserved regions.

The DNase state does not seem to follow any clear trend.

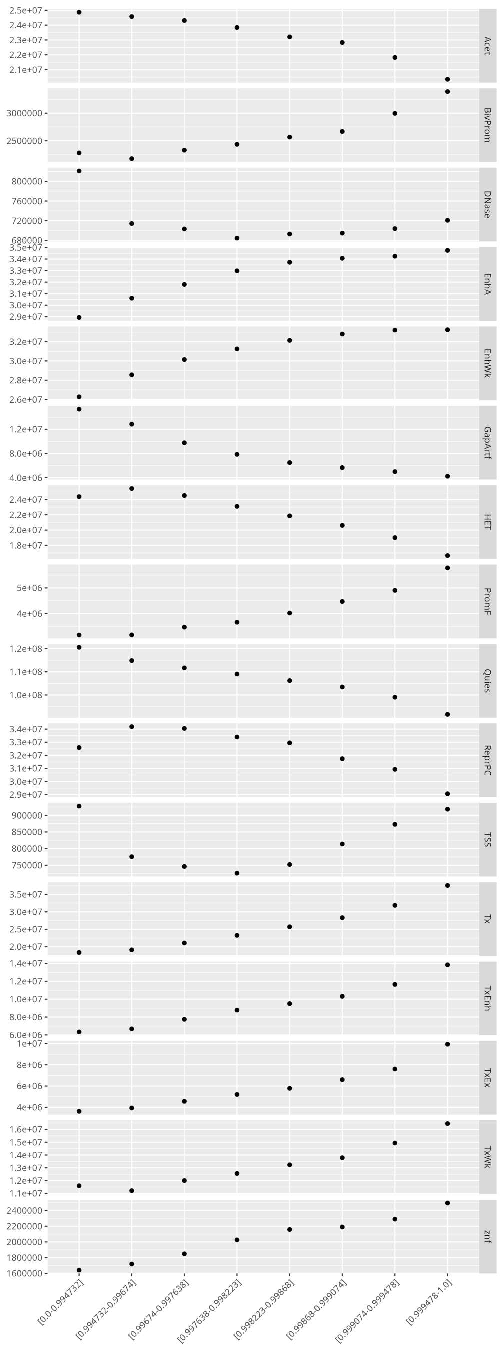

Figure 7: Results for window size of 1k.

3.2.2 Window size of 10k

Results for a window size 10k are similar to the previous ones. The correlation, however, seems slightly less clear, probably because larger windows include different types of regions.

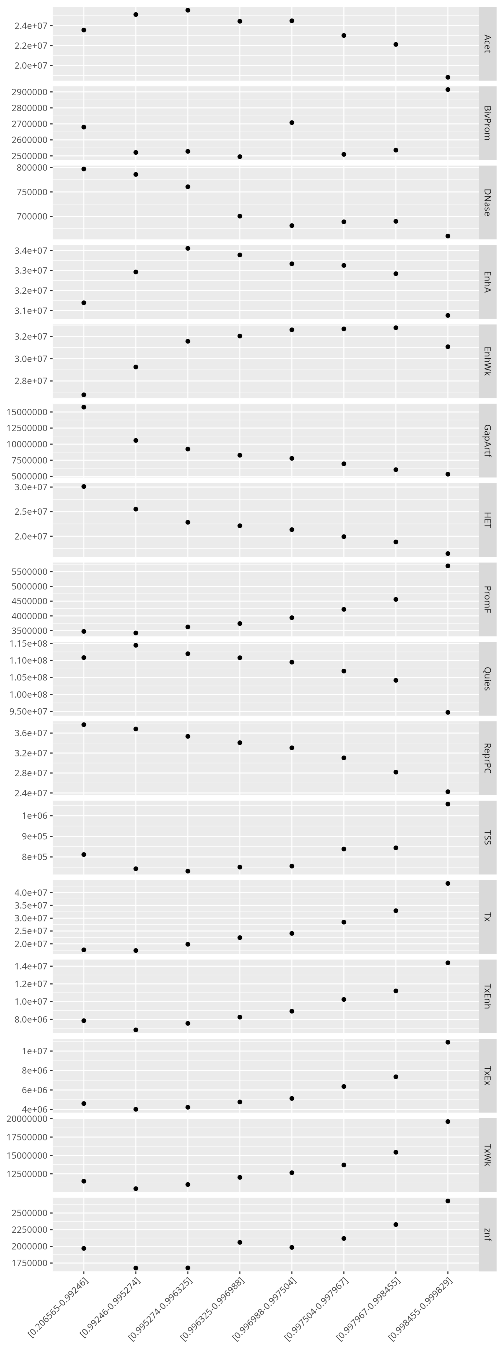

Figure 8: Results for window size of 10k.

3.2.3 Window size of 100k

Results for a window size 100k are similar to the previous ones, but even less clear.

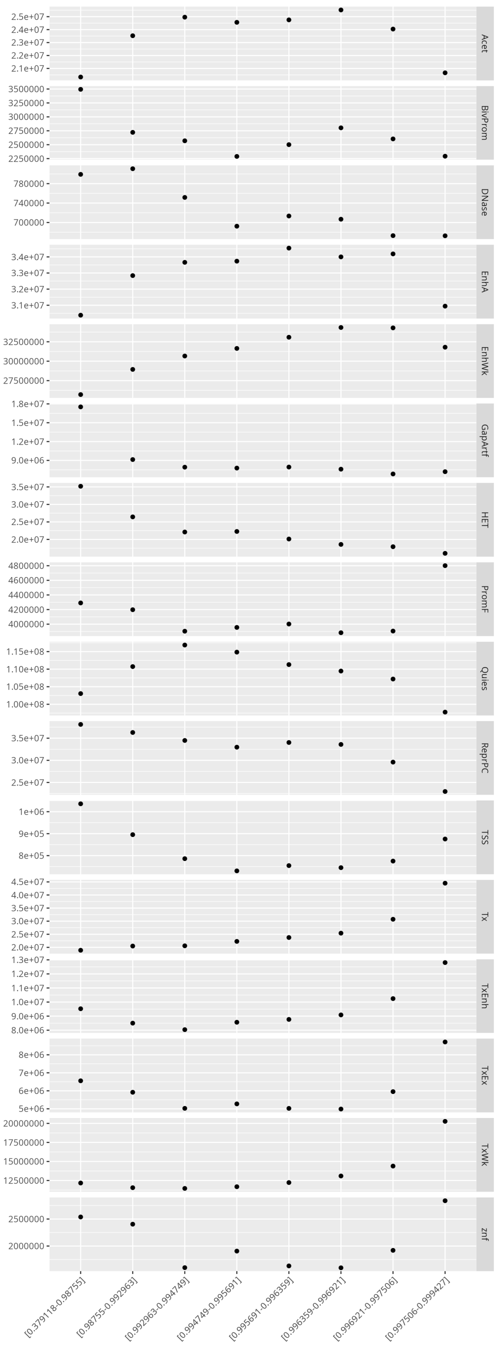

Figure 9: Results for window size of 100k.

3.2.4 Comparison with PGGB

We also run PanSel with another pangenome graph builder, PGGB. We compared the results between both tools, and the results are provided in Figure 10. The Pearson correlation is 0.84, 0.42, and 0.58 for the windows sizes of 1kb, 10kb, and 100kb respectively.

Results seem to show that MiniGraph-Cactus yields larger Jaccard indices, with many counts close to 1. A careful analysis of these differences is out of the scope of this study, but they may be explained by the number of scaffolds included in the pangenome graphs. Indeed, there are twice as many scaffolds in the PGGB graphs. As explained in [2], PGGB includes more divergent scaffolds, which MiniGraph-Cactus cannot place in the graph. These small, highly divergent, scaffolds likely decrease the local conservation where they are placed. It is however difficult to state that PGGB is more exhaustive, since placing these scaffolds is always complex, and may contain errors.

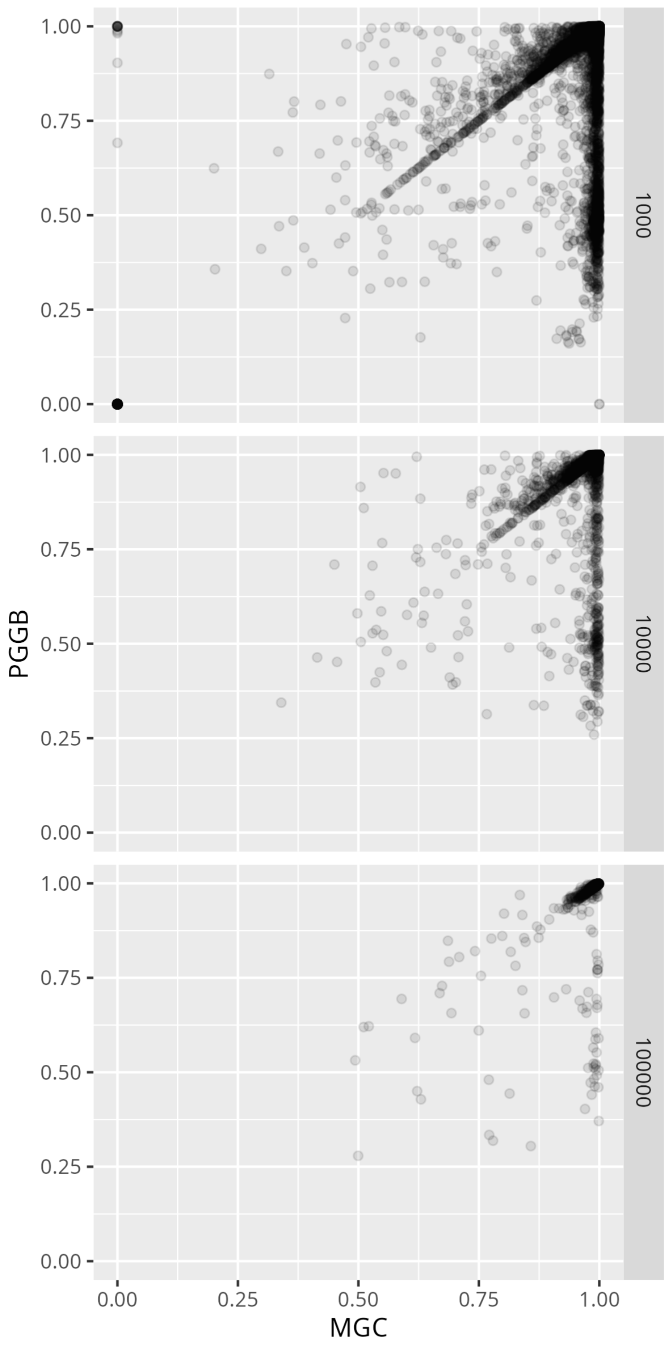

Figure 10: Correlation between MiniGraph-Cactus and PGGB results. Each dot is a window, where the x value is the PanSel score for MiniGraph-Cactus, and the y value is the Pansel score fo PGGB.

3.3 Study of particular genes

3.3.1 *ANKRD30A*

Figure 11 shows the gene *ANKRD30A* (Ankyrin Repeat Domain 30A), which has been associated with breast cancer.

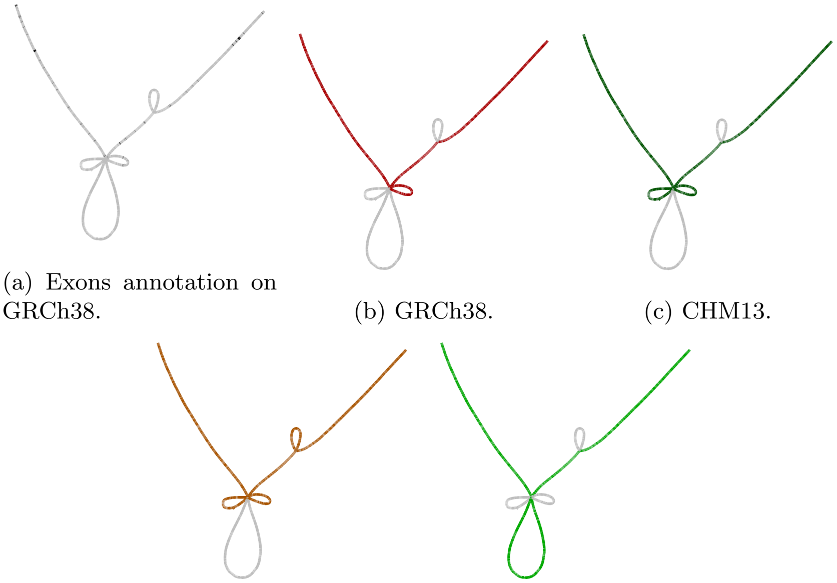

(a) Exons annotation on GRCh38.

(b) GRCh38.

(c) CHM13.

(d) HG00733.2 haplotype.

(e) HG01123.2 haplotype.

Figure 11: *ANKRD30A*: exon annotation on GRCh38, and different assemblies and paths.

3.3.2 *BRF1*

Figure 12 shows the gene *BRF1*, which is one of the subunits of the RNA polymerase III transcription factor complex.

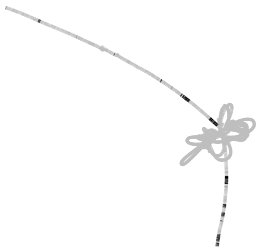

(a) Exons annotation on GRCh38.

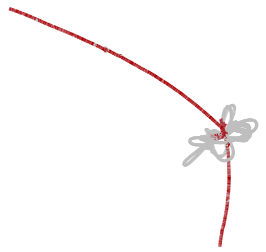

(b) GRCh38.

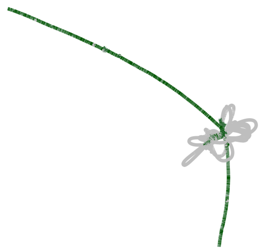

(c) CHM13.

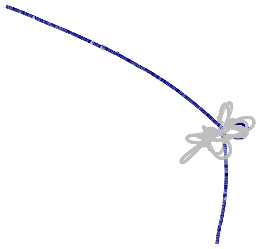

(d) HG00621.1 haplotype.

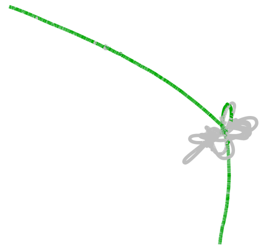

(e) HG01071.1 haplotype.

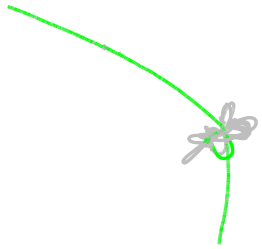

(f) HG01106.1 haplotype.

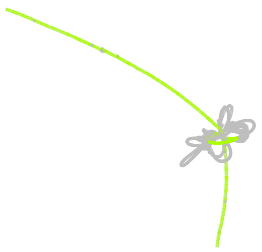

(g) HG01358.1 haplotype.

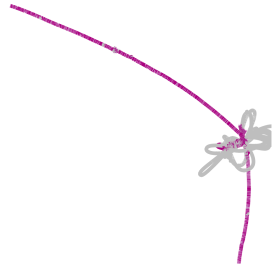

(h) HG02145.2 haplotype.

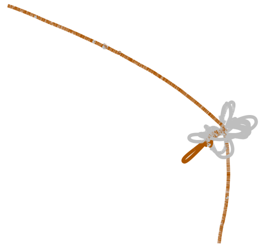

(i) NA18906.1 haplotype.

Figure 12: *BRF1*: exon annotation on GRCh38, and different assemblies and paths.

3.4 Density of conserved and divergent regions

This section provides the number of divergent and conserved regions along the chromosomes. The numbers on the y-axis gives the number of significant regions within 1Mbp. Results are given for the different window sizes.

3.4.1 Window size of 1k

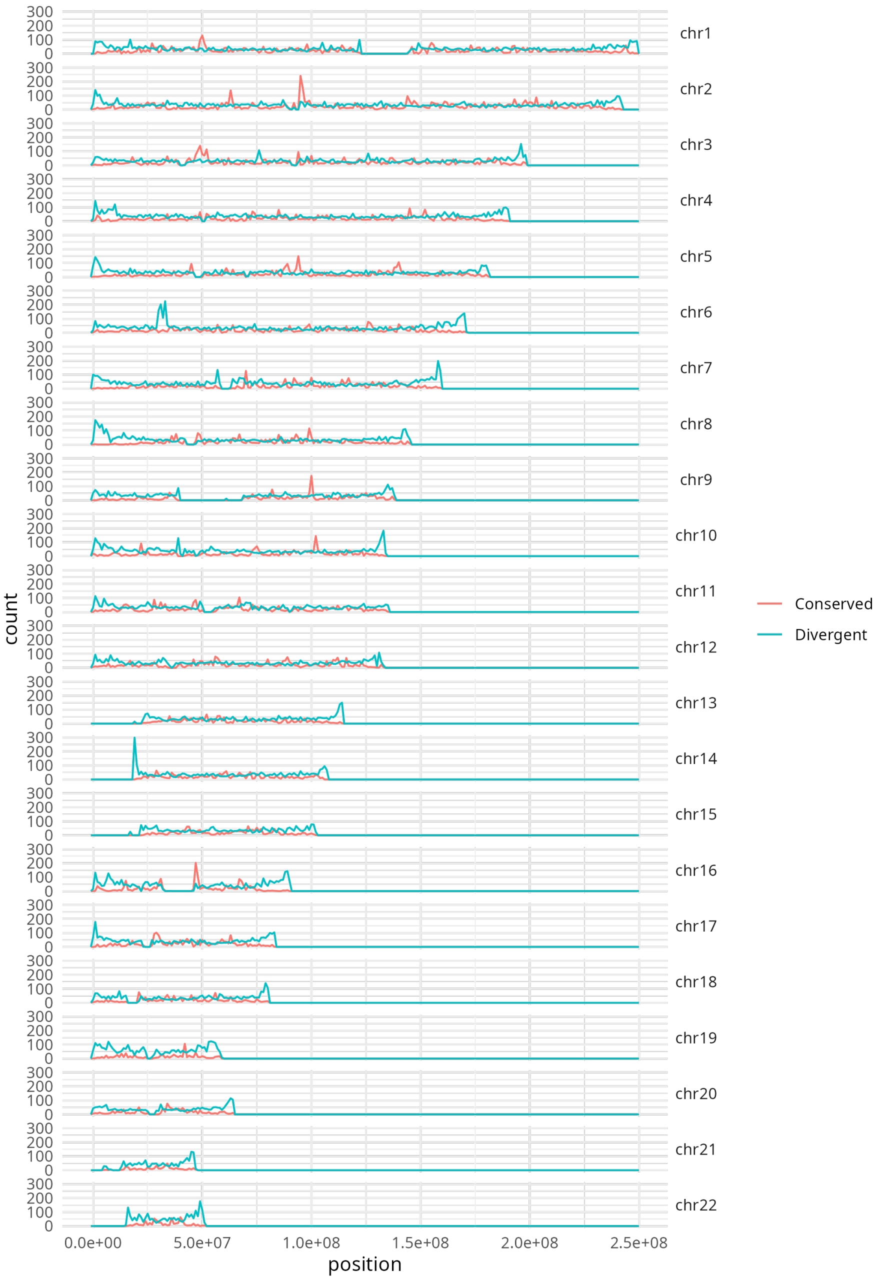

Figure 13: Density of conserved and divergent regions for window size of 1k.

3.4.2 Window size of 10k

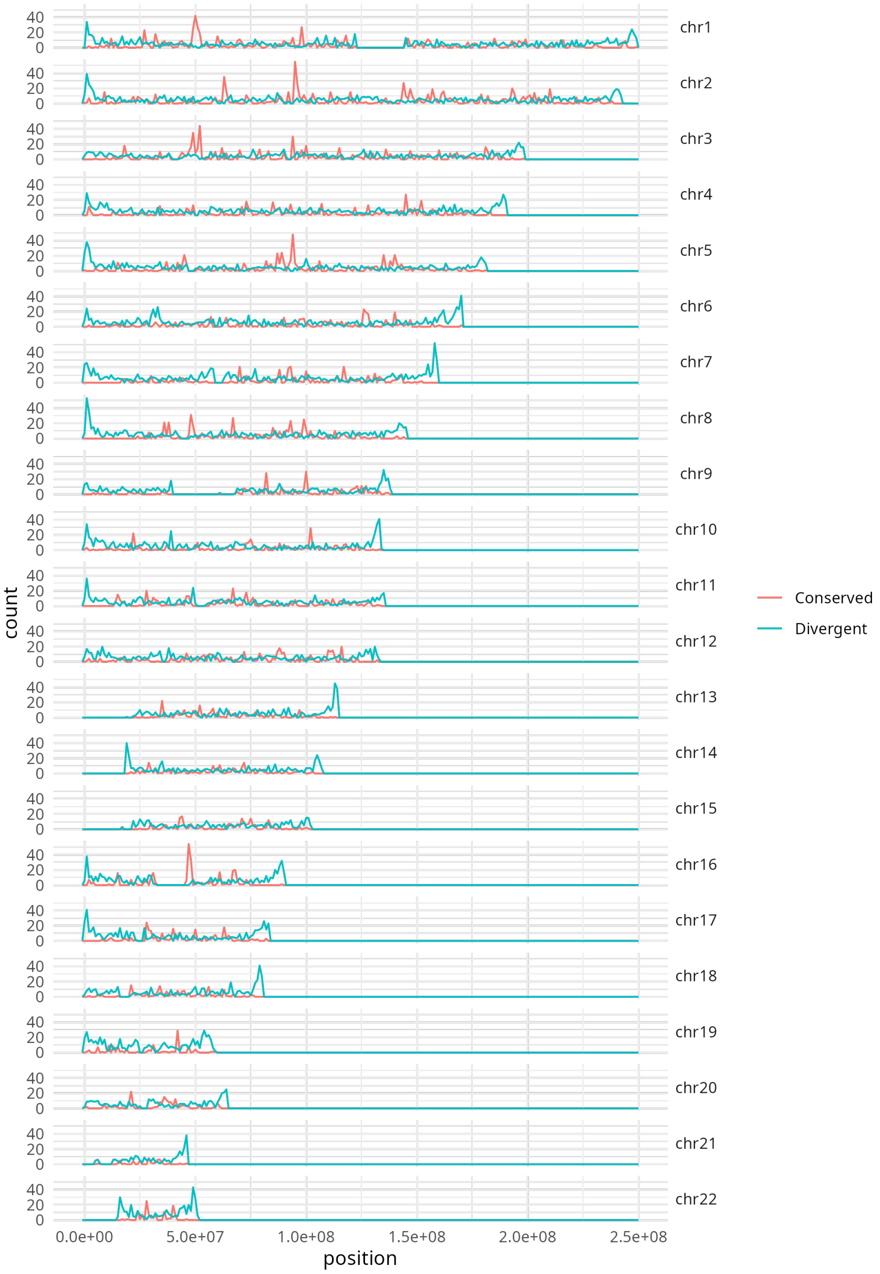

Figure 14: Density of conserved and divergent regions for window size of 10k.

3.4.3 Window size of 100k

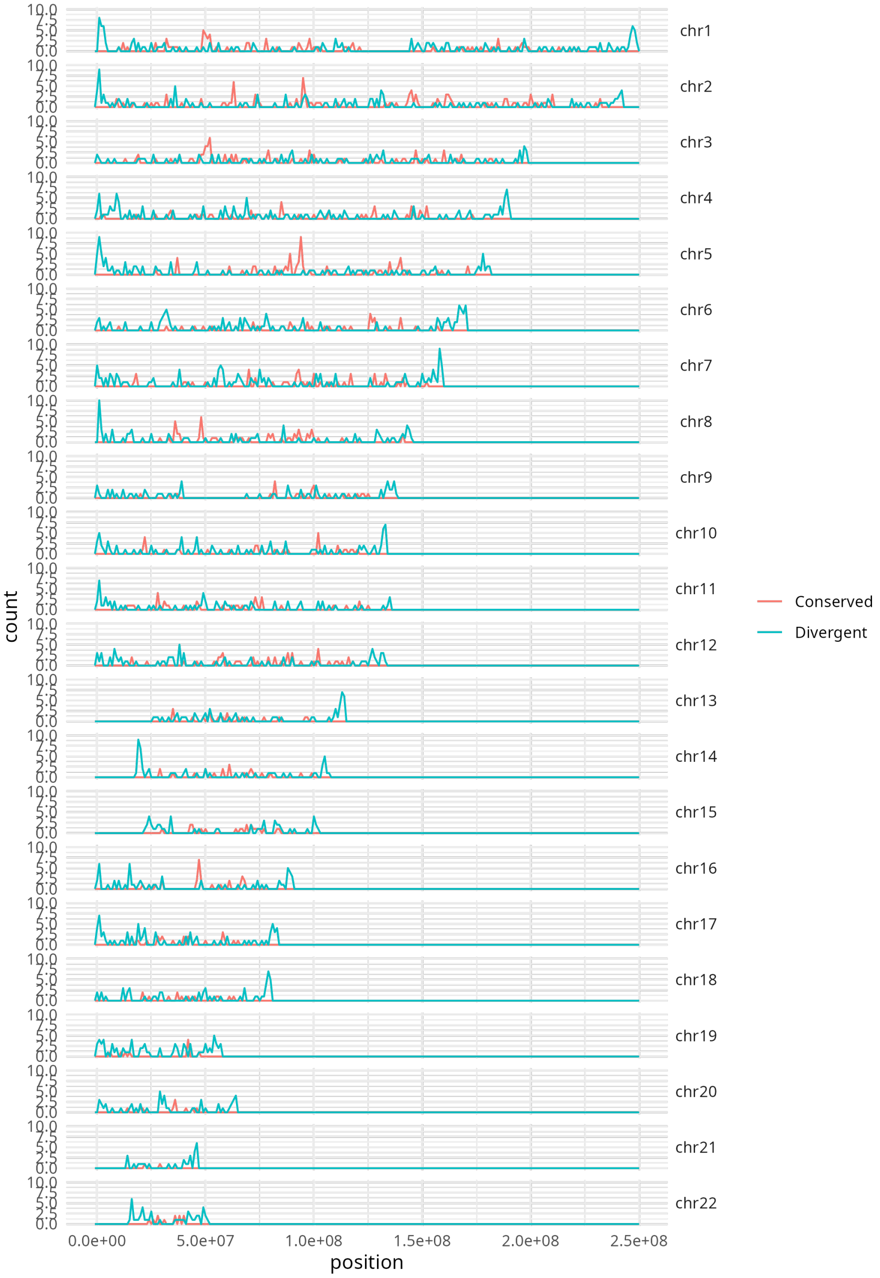

Figure 15: Density of conserved and divergent regions for window size of 100k.

3.5 Gene set enrichment

The gene set enrichment results for conserved regions are given in Figure 16. The first figure shows significantly enriched gene sets in the molecular function (MF), biological process (BP), and cellular compartment (CC) ontologies. The y-axis give the p-value.

Enriched gene sets are very vague, and shared in most species.

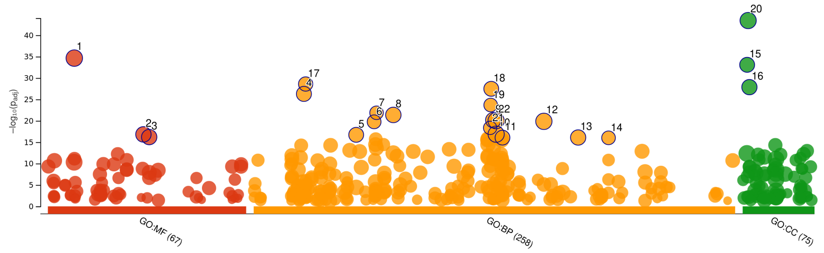

ID	Source	Term ID	Term Name	p_{adj} (query_1)
1	GO:MF	GO:0005515	protein binding	2.241×10^{-35}
2	GO:MF	GO:0036094	small molecule binding	1.531×10^{-17}
3	GO:MF	GO:0043167	ion binding	6.377×10^{-17}
4	GO:BP	GO:0007275	multicellular organism development	5.265×10^{-27}
5	GO:BP	GO:0019219	regulation of nucleobase-containing compound ...	1.923×10^{-17}
6	GO:BP	GO:0022008	neurogenesis	1.733×10^{-20}
7	GO:BP	GO:0030182	neuron differentiation	1.351×10^{-22}
8	GO:BP	GO:0032502	developmental process	4.369×10^{-22}
9	GO:BP	GO:0048856	anatomical structure development	6.960×10^{-21}
10	GO:BP	GO:0050794	regulation of cellular process	1.333×10^{-17}
11	GO:BP	GO:0051252	regulation of RNA metabolic process	8.728×10^{-17}
12	GO:BP	GO:0065007	biological regulation	1.363×10^{-20}
13	GO:BP	GO:0080090	regulation of primary metabolic process	8.104×10^{-17}
14	GO:BP	GO:0120036	plasma membrane bounded cell projection orga...	9.687×10^{-17}
15	GO:CC	GO:0005654	nucleoplasm	8.362×10^{-34}
16	GO:CC	GO:0005829	cytosol	1.371×10^{-28}
17	GO:BP	GO:0007399	nervous system development	2.552×10^{-29}
18	GO:BP	GO:0048731	system development	3.353×10^{-28}
19	GO:BP	GO:0048699	generation of neurons	2.091×10^{-24}
20	GO:CC	GO:0005737	cytoplasm	3.692×10^{-44}
21	GO:BP	GO:0048666	neuron development	4.517×10^{-19}
22	GO:BP	GO:0050789	regulation of biological process	9.429×10^{-21}

version e111_eg58_p18_f463989d
date 8/21/2024, 2:55:48 PM
organism hsapiens

g:Profiler

Figure 16: Gene Ontology results for conserved regions, computed with a window size of 10k.

The gene set enrichment results for divergent regions are given in Figure 17. Here, some genes related to immunity can be found in all three ontologies.

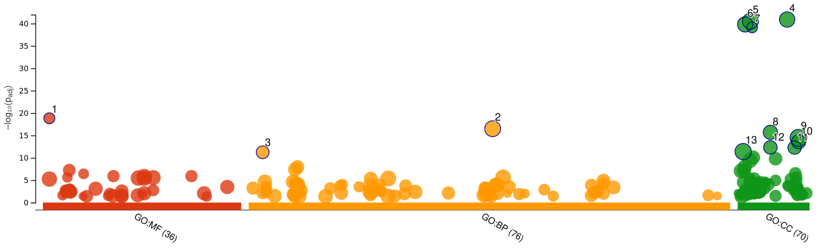

ID	Source	Term ID	Term Name	p_{adj} (query_1)
1	GO:MF	GO:0003823	antigen binding	1.491×10^{-19}
2	GO:BP	GO:0050896	response to stimulus	3.170×10^{-17}
3	GO:BP	GO:0002250	adaptive immune response	5.840×10^{-12}
4	GO:CC	GO:0071944	cell periphery	1.275×10^{-41}
5	GO:CC	GO:0016020	membrane	2.962×10^{-41}
6	GO:CC	GO:0005886	plasma membrane	1.564×10^{-40}
7	GO:CC	GO:0019814	immunoglobulin complex	7.383×10^{-40}
8	GO:CC	GO:0042995	cell projection	2.051×10^{-16}
9	GO:CC	GO:0110165	cellular anatomical entity	3.488×10^{-15}
10	GO:CC	GO:0120025	plasma membrane bounded cell projection	2.566×10^{-14}
11	GO:CC	GO:0098590	plasma membrane region	5.360×10^{-13}
12	GO:CC	GO:0043005	neuron projection	4.709×10^{-13}
13	GO:CC	GO:0005737	cytoplasm	4.147×10^{-12}

**version**	e111_eg58_p18_f463989d	g:Profiler
**date**	8/21/2024, 2:55:15 PM	
**organism**	hsapiens	

Figure 17: Gene Ontology results for divergent regions, computed with a window size of 10k.

3.6 Test on the bacteria *Myxococcus xanthus*

We use PanSel on the bacteria *M. xanthus*, a dataset provided by BubbleGun [1]. As previously, we split the windows into eight categories, from the most divergent to the most conserved. For each window, we computed the gene coverage. As seen in Figure 18, results clearly confirm that divergent regions contain less genes.

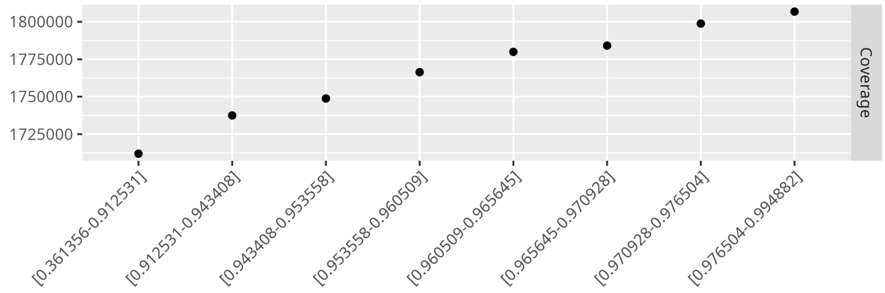

Figure 18: Distribution of the gene coverage, given the conservation.

References

[1] Fawaz Dabbaghie, Jana Ebler, and Tobias Marschall. "BubbleGun: enumerating bubbles and superbubbles in genome graphs". In: *Bioinformatics* 38.17 (2022), pp. 4217–4219. DOI: 10.1093/bioinformatics/btac448.

[2] Wen-Wei Liao et al. "A draft human pangenome reference". In: *Nature* 617.7960 (May 2023), pp. 312–324. ISSN: 1476-4687. DOI: 10.1038/s41586-023-05896-x. URL: http://dx.doi.org/10.1038/s41586-023-05896-x.
